# The effect of air pollution exposure on foetal growth restriction in pregnant women who conceived by in vitro fertilisation a cross-sectional study

**DOI:** 10.1038/s41598-025-87955-z

**Published:** 2025-01-28

**Authors:** Yanqiong Jiang, Pengfei Liu, Meihong Zeng, Guiying Zhuang, Cuiqing Qiu, Liyun Wang, Weiling Liu, Weiqi Liu

**Affiliations:** 1 Division of Neonatology, The Maternal and Children Health Care Hospital (Huzhong Hospital) of Huadu, Guangzhou, Guangdong People’s Republic of China; 2 Department of Laboratory Medicine, The Third Affiliated Hospital of Southern Medical University, Southern Medical University, Guangzhou, People’s Republic of China; 3 Medical Information Office, The Maternal and Children Health Care Hospital (Huzhong Hospital) of Huadu, Guangzhou, Guangdong People’s Republic of China; 4 Reproductive Medicine Department, The Maternal and Children Health Care Hospital (Huzhong Hospital) of Huadu, Guangzhou, Guangdong People’s Republic of China; 5 Department of Clinical Laboratory, Foshan Fosun Chancheng Hospital, Foshan, Guangdong People’s Republic of China; 6 Department of Clinical Laboratory, The Maternal and Children Health Care Hospital (Huzhong Hospital) of Huadu, Guangzhou, Guangdong People’s Republic of China

**Keywords:** Air pollutants, Foetal growth restriction, *In vitro fertilisation*, Pregnant women, Environmental sciences, Medical research, Risk factors

## Abstract

Studies investigating the relationship between exposure to air pollutants during pregnancy and foetal growth restriction (FGR) in women who conceive by in vitro fertilisation (IVF) are lacking. The objective was to investigate the effect of air pollutant exposure in pregnancy on FGR in pregnant women who conceive by IVF. We included pregnant women who conceived by IVF and delivered healthy singleton babies in Guangzhou from October 2018 to September 2023. We also collected data on air pollutant concentrations in Guangzhou during the same period. We analysed the impact of air pollution exposure during pregnancy on FGR. After adjusting for confounders, our analysis showed that in the first trimester, high concentrations of PM_10_ and NO_2_ in the fourth quartile significantly increased the risk of FGR. Specifically, the odds ratios were 6.430 (95% confidence interval [CI]: 1.035–39.96) for PM_10_ and 10.73 (95% CI: 1.230–93.48) for NO_2_. In the second trimester, exposure to PM_2.5_, PM_10_, and NO_2_ was associated with an increased risk of FGR. In addition, subgroup analyses showed that exposure to NO_2_ during pregnancy increased the risk of FGR in women aged 35 years and older. The results of this cross-sectional study suggest that exposure to PM_2.5_, PM_10_, and NO_2_ in pregnant women who conceive by IVF is associated with the occurrence of FGR.

**Supplementary Information:**

The online version contains supplementary material available at 10.1038/s41598-025-87955-z.

## Introduction

Foetal growth restriction (FGR) is a relatively common complication of pregnancy. The occurrence of FGR is associated with an increased risk of perinatal complications, increased infant morbidity and mortality^1^ and respiratory, cardiovascular and neurological problems after birth^2,3^. A retrospective study by Shankar et al.^4^ found that 50.8% of unexplained stillbirths were associated with identifiable factors, of which 41.5% were attributable to FGR. Therefore, FGR should be considered highly important by doctors and expectant mothers.

Although many factors can impact FGR risk, including maternal characteristics, lifestyle, and pregnancy complications, the relationship between exposure to air pollutants and FGR risk has not been determined^5–7^. Studies have shown that fine particulate matter (PM_2.5_) can enter the bloodstream during pregnancy and affect foetal growth^8,9^, leading to low birth weight, premature birth and reduced head circumference^10,11^. Fu et al.^12^ reported that exposure of pregnant women to high levels of nitrogen dioxide (NO_2_) and PM_2.5_ during pregnancy may affect infant head circumference and length. A study conducted in southern Sweden revealed that for every 10 µg/m^3^ increase in the NO_x_ concentration, birth weight decreased by 9 g^13^. A retrospective cohort study conducted in Australia revealed that NO_2_ exposure increases the risk of FGR by 31% (95% confidence interval [CI]: 1.07–1.60)^14^. However, some studies have shown no association between exposure to air pollutants and FGR risk. Bobak M. found no association between intrauterine growth retardation (IUGR) and exposure to air pollutants in an analysis of data from 108,173 singleton births in the Czech Republic^6^. Dejmek et al.^15^ also found no correlation between IUGR and exposure to particulate matter, sulphur dioxide (SO_2_), NOx, or ozone (O_3_). A study carried out in Norway also showed that NO_2_, PM_10_ (particles with an aerodynamic equivalent diameter ≤ 10 μm) and O_3_ had no significant effect on foetal weight or length^16^. Therefore, the relationship between air pollution exposure and FGR risk may vary according to geographical location, living standards, etc., and further research on the relationship between exposure to air pollutants and FGR risk in different regions is needed.

In recent years, the use of assisted reproductive technologies, particularly in vitro fertilisation (IVF), has increased, making it essential to understand the specific risks associated with these pregnancies. Despite the growing number of pregnancies achieved through IVF, little is known about the specific effects of exposure to air pollutants on the risk of FGR in this population. Preliminary evidence suggests that air pollution may have a more pronounced effect on pregnancies achieved through assisted reproductive technologies^17,18^, but the underlying mechanisms of these associations remain unclear.

This study aims to fill the gap by investigating the impact of exposure to air pollutants during pregnancy on the risk of FGR in pregnancies achieved through IVF. It will provide a basis for the development of targeted health policies and interventions by analysing the effects of air pollutants on the risk of FGR in pregnancies achieved through assisted reproductive technologies.

## Methods

### Participant selection

**Fig. 1:**
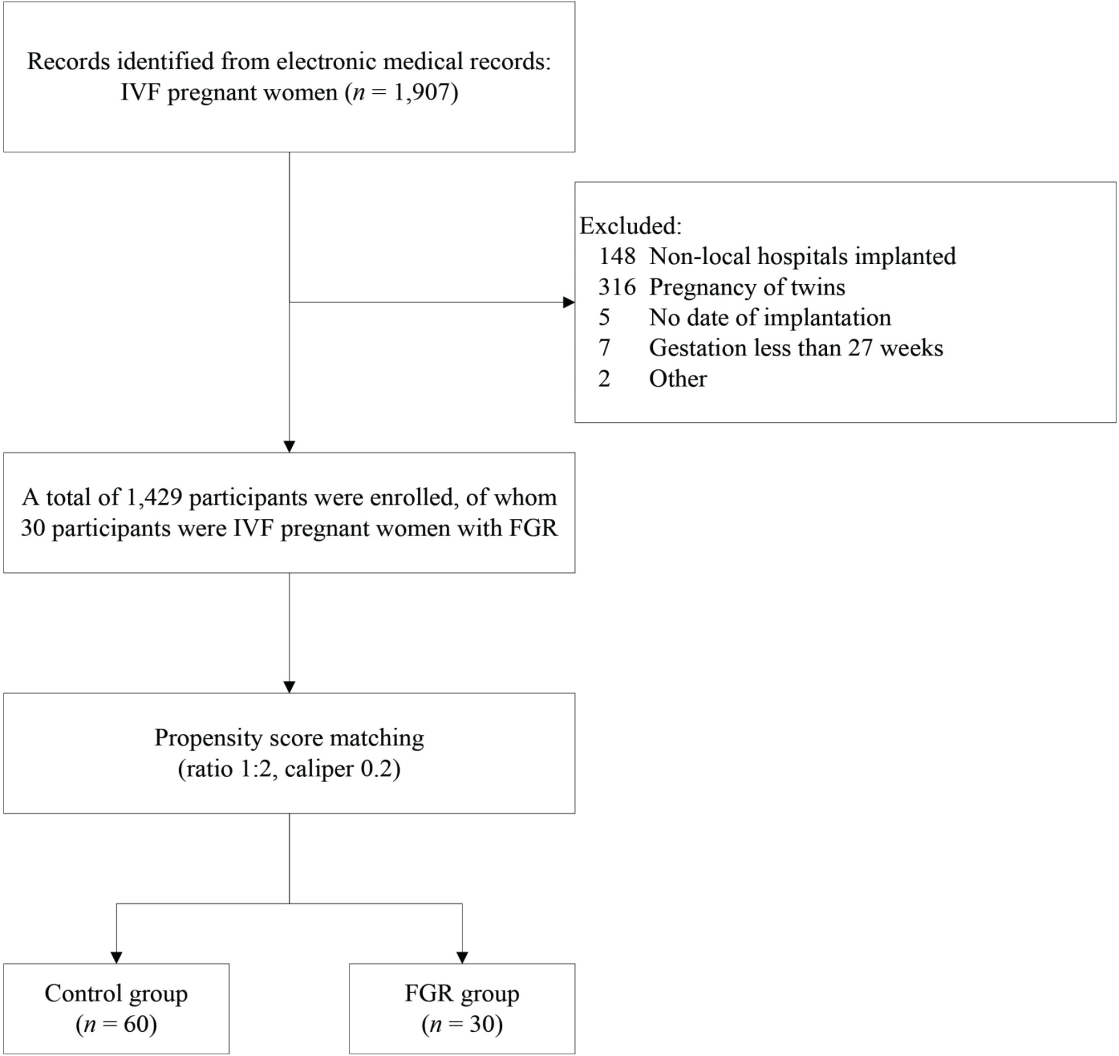
Study flowchart for selecting pregnant women who conceived by IVF. *IVF* in vitro fertilization, *FGR* Foetal growth restriction.

In this study, pregnant women who conceived by IVF and delivered in the hospital from October 2018 to September 2023 were enrolled as recruited participants using the electronic medical record information management system of Guangzhou Huadu District Maternal and Child Health Hospital. By querying the hospital’s electronic medical records, it is possible to obtain relevant information about research participants, such as age, occupation, ethnicity, residential address, implantation time, delivery date, and clinical diagnoses. The diagnosis of diseases in electronic medical records is strictly classified according to ICD-10 codes, this study included pregnant women with a diagnosis of FGR (ICD-O36.5) as the disease group, excluding pregnant women with nonlocal residential addresses and hospitals at which the embryo was transferred, those without specific implantation dates, those with twin pregnancies, gestation less than 27 weeks, and others (Fig. 1). This study was approved by the Ethics Committee of the Maternal and Children Health Care Hospital of Huadu (no. 2024-001), which waived the requirement for informed consent since the study used de-identified information. The study protocol conforms to the ethical guidelines of the 1975 Declaration of Helsinki.

### Assessment of air pollution concentrations

All those who participated in this study lived in Guangzhou. We obtained real-time concentrations of air pollutants in Guangzhou City from the National Urban Air Quality Real-Time Dissemination Platform of the China National Environmental Monitoring Centre (a pre-processed database is available at https://quotsoft.net/air, which is updated daily). In this study, we used air pollutant concentrations in Guangzhou as a proxy for individual exposure data, the time-varying average concentration method was used to estimate the mean exposure of the participants to air pollutants (PM_2.5_, PM_10_, NO_2_, O_3_) during different trimesters using real-time air pollutant concentrations in Guangzhou, based on the date of birth and the date of embryo transfer. Based on previous studies^19,20^, we calculated the average exposure concentration during three different periods: the 1st trimester (weeks 1 to 12 of pregnancy), the 2nd trimester (weeks 13 to 27) and the 3rd trimester (weeks 28 to delivery).

### Covariates and outcomes

Covariates were established based on published studies^5,13,21,22^ and information available in the hospital electronic medical record system. The covariates identified in the present study included age, occupation, ethnicity, pregnancy type, embryo type, eclampsia, anaemia, infant gender, and air pollution.

SO_2_ concentrations were obtained from the same website as PM_2.5_, PM_10_, NO_2_, and O_3_. Missing values for all air pollutant concentrations were filled in using monthly averages published by the Guangzhou Municipal Bureau of Ecology and Environment. The number of days of pregnancy varies slightly depending on the type of embryo. When transferring cleavage-stage embryo, the total number of gestation days needs to be reduced by 12 days, and when transferring blastocyst, the total number of gestation days needs to be reduced by 14 days.

FGR was diagnosed based on a clinical diagnosis obtained from the electronic medical records system. Doctors use the diagnostic criteria of the Royal College of Obstetricians and Gynaecologists for FGR^23^ and diagnose FGR when the estimated foetal weight is less than the 10th percentile for the same gestational age (SGA).

### Statistical analysis

The descriptive characteristics of the study participants were analysed using nonparametric tests for continuous variables and χ^2^ tests for categorical variables. We constructed a binary variable for FGR as the dependent variable, with the control group, air pollutants, age, occupation, pregnancy type, embryo type, eclampsia, anaemia, infant gender and SO_2_ as independent variables. To reduce the influence of baseline differences in demographic and clinical characteristics on the results, we used propensity score matching (PSM) to match the control group to the disease group in accordance with the suggestion of Lonjon et al.^24^. A logistic regression model was used to obtain the propensity score representing the likelihood of FGR. Age, occupation, ethnicity, pregnancy type, embryo type, anaemia and infant sex were used as matching factors. We then performed 1:2 greedy nearest neighbour matching with a calliper of 0.2 to match controls with normal foetal growth and development. To further assess the robustness of our findings, we conducted a sensitivity analysis using different matching ratios (1:1, 1:2, 1:3, and 1:4). After matching, conditional logit regression was used to calculate the odds ratio (OR) and 95% CI to analyse the relationship between exposure to air pollutants and FGR risk, adjusting for potential confounders. These confounders included age, occupation, pregnancy type, embryo type, anaemia, infant gender and SO_2_. In addition, we performed subgroup analyses by age to assess the association between exposure to PM_2.5_, PM_10_, NO_2_ and O_3_ during the entire gestation period and the risk of FGR in different age groups, in order to identify specific high-risk populations.

In our study, we performed PSM analysis using R version 4.2.2 and the MatchIt package and regression analysis using Stata 16.0 software. *p*-value < 0.05 was considered to indicate statistical significance.

## Results

### Characteristics of the baseline

A total of 1,907 pregnant women who underwent IVF were identified from the electronic medical records system. After excluding ineligible participants, 1,433 patients were enrolled in the study, including 30 in the FGR group and 1,429 in the control group. There were significant differences between the control group and the FGR group before PSM matching for eclampsia, gestational diabetes mellitus, hypertension in pregnancy and O_3_. After performing 1:2 PSM, the *p*-value of the baseline covariates have increased after matching, indicating that PSM effectively improved the balance of covariates between the control group and the FGR group (Table 1). As shown in Figure S1, it can be observed that the PSM sensitivity analysis using a 1:2 matching ratio demonstrates relatively stable and reasonable effect sizes for different pollutants.

**Table 1:** Demographics and characteristics of pregnant women who conceived by IVF, 2018–2023.

Characteristic	Before PSM matching			After PSM matching		
	Contrl group (*n* = 1399)	FGR group (*n* = 30)	*p*-value	Contrl group (*n* = 60)	FGR group (*n* = 30)	*p*-value
Age, *n* (%)			0.849			1.000
< 35 years	817 (58.40%)	17 (56.67%)		34 (56.67%)	17 (56.67%)	
≥ 35 years	582 (41.60%)	13 (43.33%)		26 (43.33%)	13 (43.33%)	
Occupation, *n* (%)			0.960			0.878
Employed^a^	833 (59.54%)	18 (60.00%)		37 (61.67%)	18 (60.00%)	
Other^b^	566 (40.46%)	12 (40.00%)		23 (38.33%)	12 (40.00%)	
Ethnicity, *n* (%)			0.108			1.000
Han	1,368 (97.78%)	28 (93.33%)		56 (93.33%)	28 (93.33%)	
Other^c^	31 (2.22%)	2 (6.67%)		4 (6.67%)	2 (6.67%)	
Pregnancy type, *n* (%)			0.352			1.000
Nonprimiparous	995 (71.12%)	19 (63.33%)		38 (63.33%)	19 (63.33%)	
Primiparous	404 (28.88%)	11 (36.67%)		22 (36.67%)	11 (36.67%)	
Embryo Type, *n* (%)			0.789			1.000
Cleavage-stage embryo	947 (67.69%)	21 (70.00%)		42 (70.00%)	21 (70.00%)	
Blastocyst	452 (32.31%)	9 (30.00%)		18 (30.00%)	9 (30.00%)	
Anemic, *n* (%)			0.131			1.000
No	786 (56.18%)	21 (70.00%)		42 (70.00%)	21 (70.00%)	
Yes	613 (43.82%)	9 (30.00%)		18 (30.00%)	9 (30.00%)	
Blood type, *n* (%)			0.519			0.714
Type A	404 (28.88%)	6 (20.00%)		19 (31.67%)	6 (20.00%)	
Type B	347 (24.80%)	6 (20.00%)		10 (16.67%)	6 (20.00%)	
Type O	543 (38.81%)	15 (50.00%)		26 (43.33%)	15 (50.00%)	
Type AB	105 (7.51%)	3 (10.00%)		5 (8.33%)	3 (10.00%)	
Infant gender, *n* (%)			0.218			1.000
Male	810 (57.90%)	14 (46.67%)		28 (46.67%)	14 (46.67%)	
Female	589 (42.10%)	16 (53.33%)		32 (53.33%)	16 (53.33%)	
Eclampsia, *n* (%)			< 0.001			0.001
No	1,305 (93.28%)	20 (66.67%)		56 (93.33%)	20 (66.67%)	
Yes	94 (6.72%)	10 (33.33%)		4 (6.67%)	10 (33.33%)	
Uterine scar, *n* (%)			0.596			0.488
No	1,170 (83.63%)	24 (80.00%)		44 (73.33%)	24 (80.00%)	
Yes	229 (16.37%)	6 (20.00%)		16 (26.67%)	6 (20.00%)	
Gestational diabetes mellitus, *n* (%)			0.033			0.143
No	1,073 (76.70%)	18 (60.00%)		45 (75.00%)	18 (60.00%)	
Yes	326 (23.30%)	12 (40.00%)		15 (25.00%)	12 (40.00%)	
Hypertension in pregnancy, *n* (%)			< 0.001			0.067
No	1,354 (96.78%)	25 (83.33%)		57 (95.00%)	25 (83.33%)	
Yes	45 (3.22%)	5 (16.67%)		3 (5.00%)	5 (16.67%)	
Thyroid disease in pregnancy, *n* (%)			0.265			0.469
No	1,356 (96.93%)	28 (93.33%)		58 (96.67%)	28 (93.33%)	
Yes	43 (3.07%)	2 (6.67%)		2 (3.33%)	2 (6.67%)	
Air pollution, mean (SD)						
PM_2.5_, µg/m^3^	25.35 (3.65)	26.05 (3.85)	0.287	25.93 (3.65)	26.05 (3.85)	0.871
PM_10_, µg/m^3^	45.86 (5.69)	45.96 (5.96)	0.943	45.90 (5.26)	45.96 (5.96)	0.963
NO_2_, µg/m^3^	36.27 (5.09)	37.82 (5.02)	0.108	37.13 (5.00)	37.83 (5.02)	0.638
O_3_, µg/m^3^	55.99 (6.77)	50.60 (7.52)	< 0.001	54.60 (6.15)	50.60 (7.52)	0.008
SO_2_, µg/m^3^	6.68 (0.81)	6.57 (0.75)	0.458	6.86 (1.01)	6.57 (0.75)	0.281

*IVF* in vitro fertilization, *FGR* Foetal growth restriction, *PM*_*2.5*_ particulate matter with aerodynamic diameter of ≤ 2.5 μm, *PM*_*10*_ particles matter with an aerodynamic equivalent diameter ≤ 10 μm, *NO*_*2*_ nitrogen dioxide, *O*_*3*_ ozone, *SO*_*2*_ sulphur dioxide.

^a^Civil servants, employees of enterprises and institutions.

^b^Self-employed, freelancer, farmers, unemployed individuals, students, etc.

^c^Buyi, Korean, Dong, etc.

### Relationship between exposure to air pollutants and FGR risk

Table 2 shows the relationship between each quartile of air pollution exposure in the first trimester and FGR risk. Using the first quartile as the reference value and adjusting for covariates, we observed that exposure to both PM_10_ and NO_2_ concentrations in the fourth quartile significantly increased the risk of FGR compared to the first quartile, with OR of 6.430 (95% CI: 1.035–39.96) for PM_10_ and 10.73 (95% CI: 1.230–93.48) for NO_2_, respectively. O_3_ exposure in the third and fourth quartiles compared to the first quartile was negatively associated with FGR, with OR and 95% CI of 0.071 (0.010–0.485) and 0.165 (0.029–0.943), respectively.

**Table 2:** Relationship between exposure to air pollutants and risk of FGR in the first trimester after propensity score matching.

Variable	Quartiles	Crude		Adjusted^a^	
		OR (95% CI)	*p*-value	OR (95% CI)	*p*-value
PM_2.5_	Q1 (< 20.32)	1 (Ref)		1 (Ref)	
	Q2 (20.32–25.63)	0.610 (0.114–3.256)	0.563	0.644 (0.110–3.765)	0.625
	Q3 (25.63–32.04)	1.372 (0.364–5.164)	0.640	1.980 (0.442–8.869)	0.372
	Q4 (≥ 32.04)	2.110 (0.467–9.533)	0.332	4.202 (0.640–27.59)	0.135
PM_10_	Q1 (< 37.79)	1 (Ref)		1 (Ref)	
	Q2 (37.79–44.71)	0.818 (0.197–3.391)	0.782	1.084 (0.235–5.011)	0.917
	Q3 (44.71–53.66)	0.805 (0.188–3.446)	0.770	1.106 (0.224–5.449)	0.902
	Q4 (≥ 53.66)	2.272 (0.602–8.569)	0.226	6.430 (1.035–39.96)	0.046
NO_2_	Q1 (< 23.00)	1 (Ref)		1 (Ref)	
	Q2 (23.00-32.24)	1.393 (0.288–6.728)	0.680	2.079 (0.399–10.84)	0.385
	Q3 (32.24–37.65)	2.750 (0.506–14.95)	0.241	5.563 (0.716–43.23)	0.101
	Q4 (≥ 37.65)	3.779 (0.707–20.20)	0.120	10.73 (1.230–93.48)	0.032
O_3_	Q1 (< 44.61)	1 (Ref)		1 (Ref)	
	Q2 (44.61–53.60)	0.564 (0.138–2.301)	0.425	0.417 (0.090–1.940)	0.265
	Q3 (53.60-57.18)	0.168 (0.036–0.790)	0.024	0.071 (0.010–0.485)	0.007
	Q4 (≥ 57.18)	0.278 (0.060–1.289)	0.102	0.165 (0.029–0.943)	0.043

^a^Adjusted for age, occupation, pregnancy type, embryo type, anaemia, infant gender and SO_2_.

*Q* quartiles, *FGR* Foetal growth restriction, *OR* odds ratio, *95% CI* 95% confidence interval, *PM*_*2.5*_ particulate matter with aerodynamic diameter of ≤ 2.5 μm, *PM*_*10*_ particles matter with an aerodynamic equivalent diameter ≤ 10 μm, *NO*_*2*_ nitrogen dioxide, *O*_*3*_ ozone, *Ref* Reference.

After adjusting for covariates, in the second trimester, our analysis showed a significant escalation in the risk of FGR for pregnant individuals exposed to PM_2.5_ and NO_2_ concentrations within the fourth quartile compared to the first quartile. Additionally, exposure to PM_10_ concentrations in both the second and fourth quartiles compared to the first quartile was associated with an increased risk of FGR, with OR of 5.142 (95% CI: 1.013–26.11) and 21.76 (95% CI: 2.602-182.00), respectively (Table 3). In the third trimester there is no significant association between exposure to PM_10_, NO_2_ and O_3_ and FGR. However, there is a negative association between PM_2.5_ exposure in the third quartile compared to the first quartile and FGR (Table 4).

**Table 3:** Relationship between exposure to air pollutants and risk of FGR in the second trimester after propensity score matching.

Variable	Quartiles	Crude		Adjust^a^	
		OR (95% CI)	*p*-value	OR (95% CI)	*p*-value
PM_2.5_	Q1 (< 20.78)	1 (Ref)		1 (Ref)	
	Q2 (20.78-26.00)	1.908 (0.554–6.565)	0.306	3.929 (0.869–17.76)	0.075
	Q3 (26.00-29.42)	1.009 (0.267–3.809)	0.989	1.838 (0.391–8.647)	0.441
	Q4 (≥ 29.42)	2.048 (0.546–7.686)	0.288	5.655 (1.011–31.64)	0.049
PM_10_	Q1 (< 39.66)	1 (Ref)		1 (Ref)	
	Q2 (39.66–44.81)	1.875 (0.495–7.093)	0.355	5.142 (1.013–26.11)	0.048
	Q3 (44.81–52.04)	1.648 (0.423–6.421)	0.472	4.993 (0.831–30.01)	0.079
	Q4 (≥ 52.04)	5.106 (1.133–23.01)	0.034	21.76 (2.602-182.00)	0.004
NO_2_	Q1 (< 33.24)	1 (Ref)		1 (Ref)	
	Q2 (33.24–37.13)	3.024 (0.593–15.42)	0.183	6.608 (0.762–57.32)	0.087
	Q3 (37.13–42.38)	2.009 (0.323–12.49)	0.454	9.790 (0.741–129.41)	0.083
	Q4 (≥ 42.38)	7.493 (1.200-46.78)	0.031	32.70 (2.332–458.42)	0.010
O_3_	Q1 (< 48.39)	1 (Ref)		1 (Ref)	
	Q2 (48.39–54.74)	0.591 (0.170–2.047)	0.406	0.570 (0.150–2.162)	0.408
	Q3 (54.74–61.15)	0.279 (0.077–1.006)	0.051	0.349 (0.079–1.539)	0.165
	Q4 (≥ 61.15)	0.653 (0.161–2.650)	0.551	0.713 (0.139–3.664)	0.686

^a^Adjusted for age, occupation, pregnancy type, embryo type, anaemia, infant gender and SO_2_.

*Q* quartiles, *FGR* Foetal growth restriction, *OR* odds ratio, *95% CI* 95% confidence interval, *PM*_*2.5*_ particulate matter with aerodynamic diameter of ≤ 2.5 μm, *PM*_*10*_ particles matter with an aerodynamic equivalent diameter ≤ 10 μm, *NO*_*2*_ nitrogen dioxide, *O*_*3*_ ozone, *Ref* Reference.

**Table 4:** Relationship between exposure to air pollutants and risk of FGR in the third trimester after propensity score matching.

Variable	Quartiles	Crude		Adjust^a^	
		OR (95% CI)	*p*-value	OR (95% CI)	*p*-value
PM_2.5_	Q1 (< 18.13)	1 (Ref)		1 (Ref)	
	Q2 (18.13–25.12)	0.428 (0.115–1.602)	0.208	0.390 (0.097–1.577)	0.187
	Q3 (25.12–34.29)	0.102 (0.018–0.575)	0.010	0.126 (0.019–0.847)	0.033
	Q4 (≥ 34.29)	0.314 (0.072–1.369)	0.123	0.634 (0.071–5.677)	0.683
PM_10_	Q1 (< 33.99)			1 (Ref)	
	Q2 (33.99–44.25)	0.621 (0.190–2.032)	0.431	0.647 (0.178–2.350)	0.508
	Q3 (44.25–55.30)	0.104 (0.017–0.630)	0.014	0.167 (0.025–1.116)	0.065
	Q4 (≥ 55.30)	0.274 (0.064–1.165)	0.080	0.742 (0.055–9.944)	0.822
NO_2_	Q1 (< 30.14)	1 (Ref)		1 (Ref)	
	Q2 (30.14–35.06)	0.737 (0.197–2.752)	0.650	0.872 (0.213–3.573)	0.849
	Q3 (35.06–43.75)	0.425 (0.104–1.745)	0.235	0.653 (0.136–3.128)	0.594
	Q4 (≥ 43.75)	0.291 (0.068–1.243)	0.096	0.880 (0.094–8.228)	0.911
O_3_	Q1 (< 26.80)	1 (Ref)		1 (Ref)	
	Q2 (44.17–50.68)	2.028 (0.460–8.936)	0.350	1.739 (0.367–8.247)	0.486
	Q3 (50.68–59.82)	0.405 (0.083–1.976)	0.264	0.345 (0.064–1.863)	0.216
	Q4 (≥ 59.82)	1.047 (0.231–4.759)	0.952	1.055 (0.205–5.416)	0.949

^a^Adjusted for age, occupation, pregnancy type, embryo type, anaemia, infant gender and SO_2_.

*Q* quartiles, *FGR* Foetal growth restriction, *OR* odds ratio, *95% CI* 95% confidence interval, *PM*_*2.5*_ particulate matter with aerodynamic diameter of ≤ 2.5 μm, *PM*_*10*_ particles matter with an aerodynamic equivalent diameter ≤ 10 μm, *NO*_*2*_ nitrogen dioxide, *O*_*3*_ ozone, *Ref* Reference.

### Subgroup analysis after PSM

**Fig. 2:**
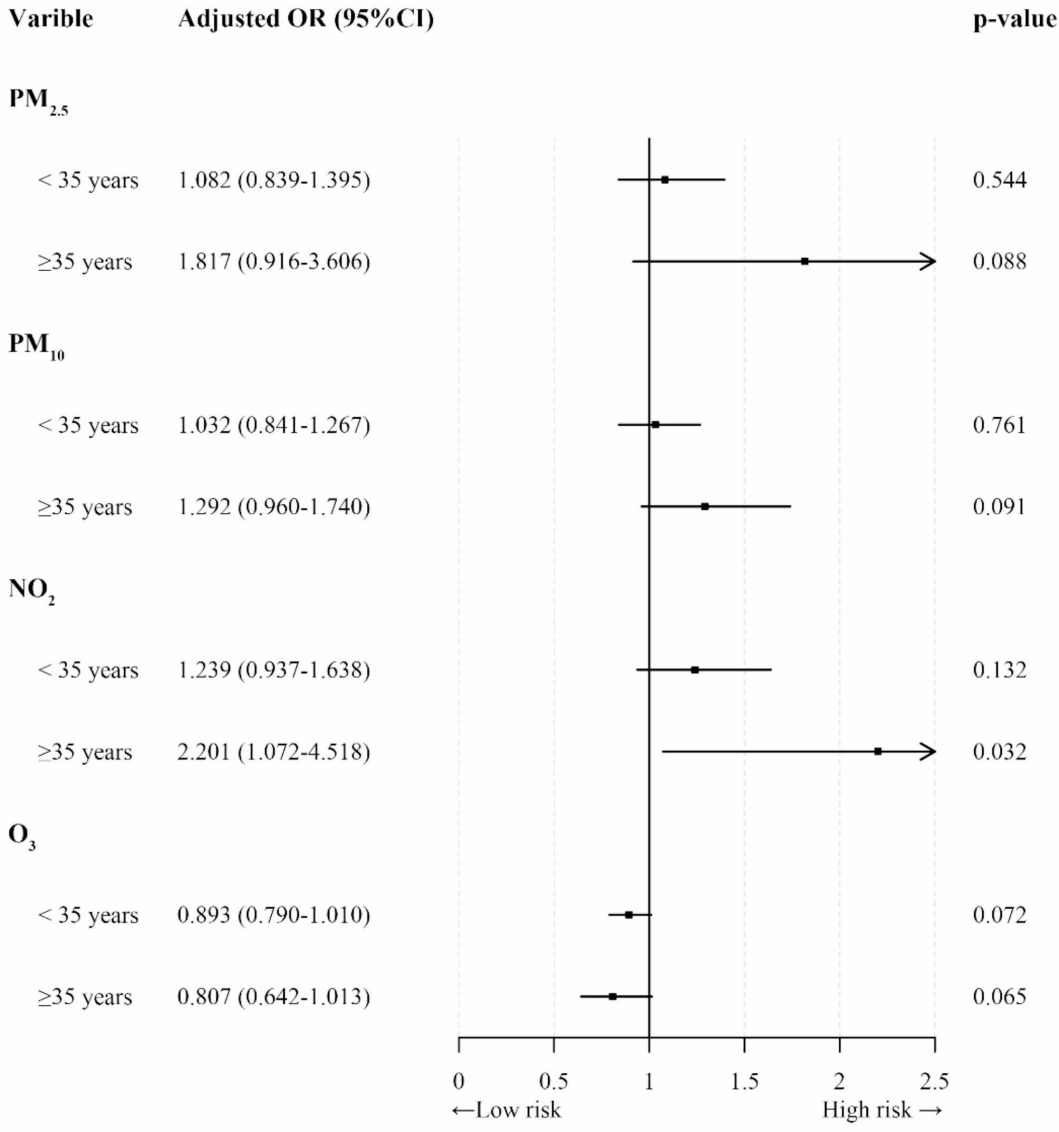
shows the association between exposure to PM_2.5_, PM_10_, NO_2_ and O_3_ during pregnancy and the risk of FGR in pregnant women of different ages. After adjusting for covariates, for pregnant women aged ≥ 35 years who were exposed to NO_2_, increased the risk of FGR (OR = 2.201; 95% CI: 1.072–4.518).

Figure 2. The association between exposure to air pollution during pregnancy and FGR in pregnant women of different ages. Adjusted for age, occupation, pregnancy type, embryo type, anaemia, infant gender and SO_2_. *FGR* Foetal growth restriction, *OR* odds ratio, *95% CI* 95% confidence interval, *PM*_*2.5*_ particulate matter with aerodynamic diameter of ≤ 2.5 μm, *PM*_*10*_ particles matter with an aerodynamic equivalent diameter ≤ 10 μm, *NO*_*2*_ nitrogen dioxide, *O*_*3*_ ozone.

## Discussion

In this cross-sectional study, we investigated the association between PM_2.5_, PM_10_, NO_2_, O_3_ exposure and FGR risk in Guangzhou, Guangdong, China. After baseline matching of the study participants at a 1:2 ratio via PSM, we found that exposure to PM_10_ and NO_2_ during the first trimester and exposure to PM_2.5_, PM_10_ and NO_2_ during the second trimester were associated with an increased risk of FGR. Specifically, exposure to NO_2_ during pregnancy was found to increase the risk of FGR in women aged ≥ 35 years. This study indicating a dose‒response relationship between adverse effects of air pollution and FGR.

There has been some controversy about the relationship between the concentration of air pollutants and FGR. PM_2.5_ exposure in entire pregnancy was strongly associated with smaller gestational age (OR = 1.15, 95%CI: 1.00-1.31) in pregnant women at Rhode Island’s Women and Infants Hospital between 2001 and 2012 ^25^, which is closely linked to FGR^26^. A Utah-based study of 51,086 mothers found that exposure to PM_10_ and NO_2_ in the first, second and third trimester of pregnancy associated with increased risk of FGR^27^. In addition, a study on the impact of exposure to air pollutants on foetal growth revealed that exposure to PM_10_ and NO_2_ during the third trimester was negatively associated with head circumference at birth, and NO_2_ exposure was negatively associated with birth length in the first, second, and third trimesters^28^. A previous French study showed that a 10 µg/m^3^ increase in NO_2_ exposure during the second trimester, the risk of FGR increased by 55% (95% CI: 1.06–2.27), but exposure to NO_2_ during early and third trimester does not increase the risk of^29^. However, a study conducted in Seoul, South Korea, of 824,011 singleton full-term newborns showed that the correlation between PM_2.5_ and PM_10_ exposure and FGR was close to zero^30^. A study in Nigeria revealed that PM_2.5_ exposure had no effect on foetal growth^31^. In this study, we found that exposure to PM_10_ and NO_2_ during the first trimester was positively correlated with an increased risk of FGR. Furthermore, exposure to PM_2.5_, PM_10_, and NO_2_ in the second trimester was also associated with a heightened risk of FGR. However, no significant differences were observed in the relationship between air pollutant exposure and FGR during the third trimester pregnancy. The difference in results may be due to the different geographical setting and occurrence of air pollution exposure during pregnancy in our study compared with studies in Rhode Island, Utah, Seoul and Nigeria. In addition, our study found significant effects mainly in early and mid-pregnancy, which differs from other studies that reported effects throughout pregnancy. This may be because early and mid-pregnancy are critical periods of foetal development when key organs and systems are being formed, making the foetus more sensitive to environmental factors. In addition, during these early stages, the functions and barriers of the placenta are not fully developed, potentially allowing more pollutants to affect the foetus. Finally, differences in sample size, population characteristics and study design (including measurement methods and data analysis) may also lead to inconsistent results.

Studies have showed that air pollutants can cause histopathological changes in the placenta^32^, increased oxidative stress^33^, and placental DNA methylation^34^, leading to impaired placental function, which in turn allows air pollutants to affect foetal growth and development through the placenta^35,36^. The results of the Early Autism Risk Longitudinal Investigation from the Infinium HumanMethylation450k platform for 133 placenta platforms showed that prenatal exposure to O_3_ can cause placental methylation and affect foetal growth and development^37^. A study conducted in Shanghai from 2015 to 2018 found that exposure to PM_2.5_ and O_3_ during pregnancy reduced foetal femur length and head circumference at 36 weeks’ gestation^38^, this finding suggests that exposure to O_3_ during pregnancy may have effects on foetal development. However, these previous findings are not entirely consistent with the results of this study. This study found a negative association between O_3_ exposure in the first trimester and FGR. Additionally, there was a negative association between PM_2.5_ exposure in the third trimester and FGR. This finding is consistent with a study conducted in Canada from 1985 to 2000, which examined the relationship between air pollutants and intrauterine growth restriction in single live births at term and found that exposure to O_3_ in first, second and third trimester was negatively correlated with FGR^39^. In addition, a previous study by Wang et al.^20^ from 2015 to 2017 in Guangzhou also found a negative association between PM_2.5_ exposure in the third trimester and SGR, which is now synonymous with intrauterine or FGR^40^. Inconsistencies in research findings on the relationship between PM_2.5_ and O_3_ exposure and FGR may be due to several factors, including differences in the methods used to assess exposure in different studies; differences in the health status, lifestyle and regional characteristics of the study populations; and the influence of regional air pollution levels and other environmental factors, such as climate and lifestyle habits. Since it is logically implausible that PM_2.5_ and O_3_ exposure would reduce the risk of FGR, future steps should focus on increasing the sample size to minimise bias and conducting comparative studies in different regions and environmental settings to validate the relationship between PM_2.5_ and O_3_ exposure and FGR.

In this study, we also analysed the effect of exposure to air pollution during pregnancy on FGR risk among pregnant women of different ages. The results showed that exposure to NO_2_ during pregnancy significantly increased the risk of FGR in pregnant women aged ≥ 35 years. This may be related to the gradual decline in the physical functions (such as placental function and the immune system) of pregnant women with age, which increases their vulnerability to external environmental factors. On the other hand, their older age has caused certain changes in their immune system, resulting in increased sensitivity to air pollutants and greater vulnerability to the adverse effects of air pollutants.

Our study has several advantages. First, we used the PSM method to process the data. PSM has unique practicality and simplicity^41^ and eliminates confounding bias in the cohort when randomization methods cannot be used. Second, we used the implantation date of pregnant women who conceived by IVF as the starting time for evaluating air pollutant exposure, which more accurately reflects the actual start time of pregnancy than the last menstrual period and can more accurately reflect the exposure of pregnant women to air pollutants. This study has several limitations. First, we collected data on potential confounders by reviewing electronic medical records, however, several important factors (such as smoking^42^ and drinking status^43^) that may affect the relationship between exposure to air pollutants and FGR risk were not included in the analysis, which may bias the results. Second, we used the address registered in the hospital electronic medical records information system at the time of delivery to assess the pregnant women’s exposure to air pollutants. The lack of potential relocation information for the study participants during the study period may have led to inaccurate assessments of air pollutant exposure. Finally, exposure to air pollutants can cause oxidative stress in pregnant women^44^, while antioxidants such as tocopherols, ascorbic acid and carotenoids in food are natural antioxidants that can ameliorate oxidative stress in the body^45^. In this study, we did not collect information on the participants’ diets and were unable to determine the effect of dietary factors on the results. Dietary factors during pregnancy should be considered in future studies to better determine the effects of exposure to air pollution on FGR risk.

## Conclusion

The findings confirmed that exposure to PM_10_ and NO_2_ during the first trimester and to PM_2.5_, PM_10_ and NO_2_ during the second trimester may increase the risk of FGR in pregnant women undergoing IVF. This risk is particularly pronounced in women aged ≥ 35 years exposed to NO_2_ during pregnancy. Although this study has several limitations, the research showed that exposure to air pollution in the first and second trimester can increase the risk of FGR, which is not only associated with a higher risk of stillbirth and neonatal mortality^46,47^, but also increases the risk of metabolic syndrome in adulthood^48,49^. Considering the health implications of FGR, the actual consequences of air pollution exposure may be more severe than currently documented, making this an important area for further investigation.

## Electronic supplementary material

Below is the link to the electronic supplementary material.

Supplementary Material 1

## Data Availability

The data that support the findings of this study are available from correspondence author but restrictions apply to the availability of these data, which were used under license for the current study, and so are not publicly available. Data are however available from the authors upon reasonable request and with permission of correspondence author.
