# Supplementary material for: The effect of air pollution exposure on foetal growth restriction in pregnant women who conceived by in vitro fertilisation a cross-sectional study
Source: Sci Rep. 2025 Jan 28;15:3497. doi: 10.1038/s41598-025-87955-z (PMC11775239; doi:10.1038/s41598-025-87955-z)
Supplement: Supplementary file 1 — Supplementary Material 1 [file 41598_2025_87955_MOESM1_ESM.docx]

**Supplementary Figure**


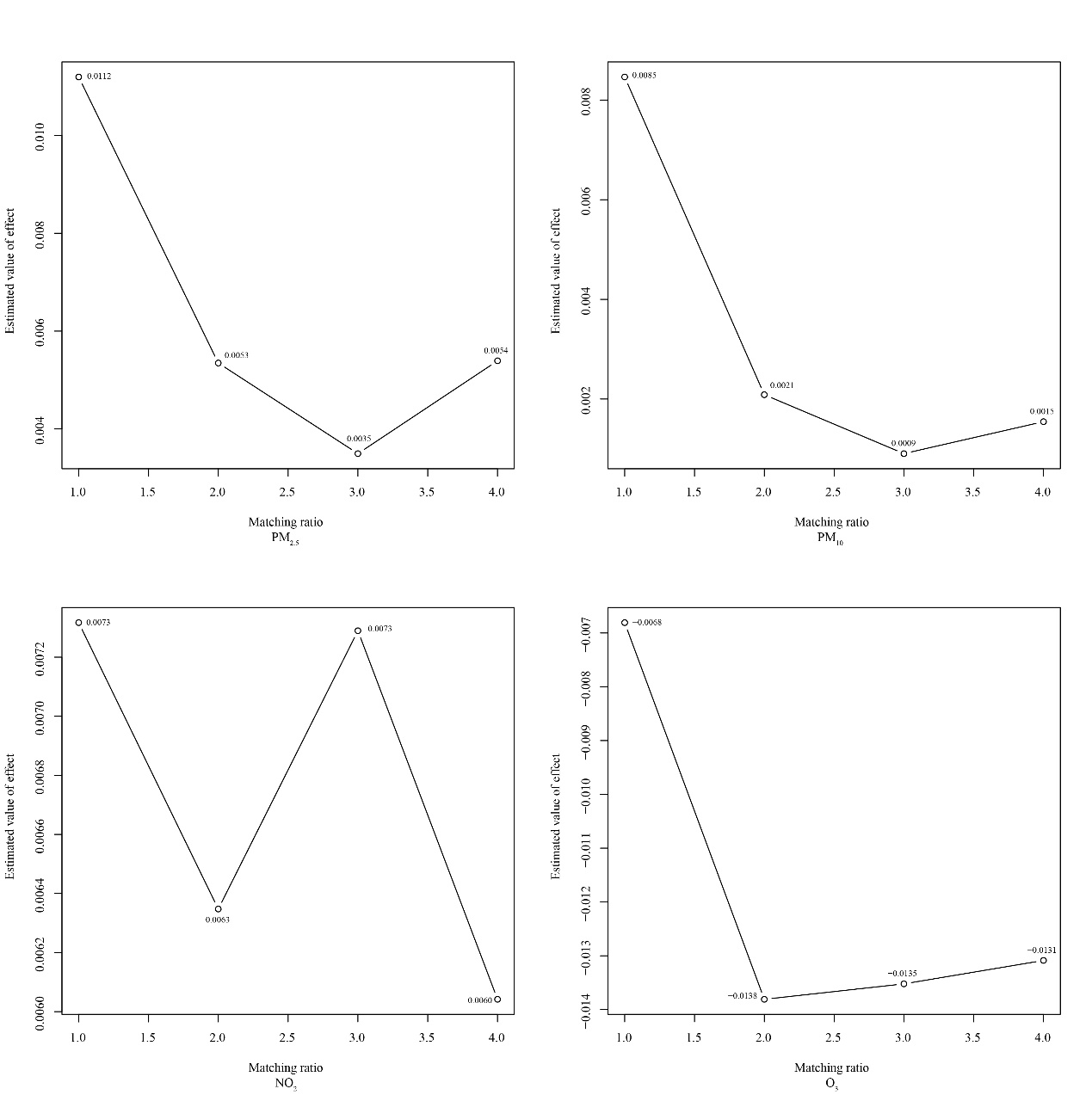


**Figure S1** Stability analysis of the effect of PM_2.5_, PM_10_, NO_2_, O_3_ on FGR with different matching ratios. *PM_2.5_*, particulate matter with aerodynamic diameter of ≤2.5μm. *PM_10_*, particles matter with an aerodynamic equivalent diameter ≤10µm. *NO_2_*, nitrogen dioxide. *O_3_*, ozone.
